# Cardiopulmonary Physical Therapist; A rescuer for the patients suffering from COVID-19

**DOI:** 10.12669/pjms.36.5.2880

**Published:** 2020

**Authors:** Malik Muhammad Ali Awan, Sidra Qureshi, Kiran Khushnood

**Affiliations:** 1 Malik Muhammad Ali Awan, DPT, MS-CPPT. Assistant Professor, Cardiovascular & Pulmonary Physical Therapy Institute of Rehabilitation Sciences, Isra University, Islamabad, Pakistan; 2 Sidra Qureshi, DPT, MS-WHPT. Demonstrator / Physical Therapist, nstitute of Rehabilitation Sciences Foundation University, Islamabad, Pakistan; 3 Kiran Khushnood, DPT, MS-NMPT. Assistant Professor, Institute of Rehabilitation Sciences Isra University, Islamabad, Pakistan

According to World Health Organization (WHO) the ailments caused by viruses have always emerged in the past and posed masses to life threatening medical conditions. Considering the last two decades, a number of viral infections emerged as epidemic including severe acute respiratory syndrome coronavirus (SARS-CoV), H1N1 influenza, Middle East respiratory syndrome coronavirus (MERS-CoV) and when the timeline approaches the present days there is an outbreak of coronavirus once again with drastic dissemination all across the globe but this time it is the novel one, having a new strain and originating from China in the city of Wuhan with its 1^st^ case reported in the month of December, 2019. The Chinese Center of Disease Control and Prevention (CDC) executed an intensive investigation program. WHO named this novel strain of coronavirus as sever acute respiratory syndrome coronavirus 2 (SARS-CoV-2) aka coronavirus disease 2019 (COVID 19). The sufferer of this virus predominantly and typically shows the symptoms of acute respiratory infections ranging from barely noticeable cough and dyspnea to need of mechanical ventilation in excessively compromised individuals but it typically causes pneumonia thus producing pneumonia like symptoms.^1^,^2^ The most common complications associated with COVID 19 is acute respiratory distress syndrome (ARDS) that include signs of pneumonia and sepsis.^3^ Pneumonia is a deadly condition and is the 8^th^ leading cause of death in USA.^4^ Physical therapists with specialized degree in cardiopulmonary physical therapy are an integral part of any healthcare organization around the globe as they deal with the patients chiefly in the acute care settings. The literature suggests that patients suffering from pneumonia or having pneumonia like symptoms admitted in hospitals benefit excessively from cardiopulmonary physical therapy as the trained professionals help them in removing the excessive thick and tenacious secretions that are the precursor of pneumonia and pneumonia in return is a deadly complication in the patients suffering from COVID 19, moreover and along with the complication of pneumonia the patients are also ridden to the beds and early mobilization out of the bed which is in the domain of physical therapy also aid in the patients general health status by reducing the length of hospital stay.^5^,^6^ Thus we can say that the physiotherapists specialized in cardiopulmonary physical therapy are a life saver and rescuer for patients having COVID 19. Recently the authors noticed that in our country Pakistan there are some misconceptions among the healthcare professionals regarding the role of physical therapy in COVID 19, stating little or simply no role of a physical therapist in the said disease. So, now it is clarified in the light of supported evidence that, in addition to other expert healthcare professionals, physical therapists are key personnel in the management of coronavirus disease 2019.

## Authors’ Contribution

**MMAA:** Concept, design and accountable for all work.

**SQ:** Drafting and revision.

**KK:** Critical analysis and final approval.
